# Complications and Recurrence of Patellar Instability after Medial Patellofemoral Ligament Reconstruction in Children and Adolescents: A Systematic Review

**DOI:** 10.3390/children8060434

**Published:** 2021-05-21

**Authors:** Riccardo D’Ambrosi, Katia Corona, Paolo Capitani, Gianluca Coccioli, Nicola Ursino, Giuseppe Maria Peretti

**Affiliations:** 1 IRCCS Istituto Ortopedico Galeazzi, Via Galeazzi 4, 20161 Milan, Italy; gianluca.coccioli@unimi.it (G.C.); nicolaursino@libero.it (N.U.); giuseppe.peretti@unimi.it (G.M.P.); 2 Dipartimento di Medicina e Scienze Della Salute Vincenzo Tiberio, Università degli Studi del Molise, 86100 Campobasso, Italy; katia.corona@unimol.it; 3 A.S.S.T. Grande Ospedale Metropolitano Niguarda, Piazza dell’Ospedale Maggiore 3, 20162 Milan, Italy; paolocapitani.dr@gmail.com; 4 Dipartimento di Scienze Biomediche per la Salute, Università degli Studi di Milano, Via Mangiagalli 31, 20133 Milan, Italy

**Keywords:** medial patellofemoral ligament, patellar instability, anterior knee pain

## Abstract

Background: This study aimed to review the data available in the current literature concerning the complications and recurrence of instability following medial patellofemoral ligament (MPFL) reconstruction for patellar instability in young and adolescent patients (those <20 years old). Methods: A systematic review was performed based on the preferred reporting items for systematic reviews and meta-analyses (PRISMA) guidelines. Two independent reviewers searched the PubMed, Scopus, EMBASE, and Cochrane databases. The terms “medial patellofemoral ligament” or “MPFL” and “reconstruction” and “young” or “adolescents” or “children” were used. The inclusion criteria for the literature review comprised studies that reported the complications and recurrences of instability in patients who had undergone MPFL reconstruction for patellar instability. Results: In all, 332 patients were included in the review, of which 195 were females (63.5%) and 112 were males (36.5%), and they totaled 352 treated knees. The mean age at the time of the surgery was 14.28 years, and the mean follow-up duration was 30.17 months. A total of 16 (4.5%) complications were reported: one (0.3%) patella fracture, one (0.3%) screw removal due to intolerance, one (0.3%) infection, five (1.4%) wound complications, six (1.7%) subluxations and two (0.6%) instances of post-operative stiffness. A total of 18 (5.1%) recurrences of patellar instability were recorded. Conclusions: MPFL reconstruction in young patients can be considered an effective and safe treatment leading to clinical improvement in terms of recurrence of dislocation. No major complications related to the technique were reported, but a high level of research evidence is required to better evaluate the clinical results in a long-term follow-up.

## 1. Introduction

Patellar dislocation is one of the most recognized acute knee injuries in young and active patients. Conservative treatment is suggested after first-time patellar dislocation without loose osteochondral fragments including activity modification, patellar bracing, and physical therapy focused on quadriceps and hip abductor strengthening [1].

Medial patellofemoral ligament (MPFL) reconstruction may be indicated for young patients with recurrent patellar instability [2]. Chronic patellar instability episodes may lead to further cartilage injury and debilitating pain, limit daily living activities, and stall one’s return to sports [3].

Although MPFL is disrupted after acute patellar dislocations in 94% of the patients, a multitude of factors can contribute to patellofemoral instability. These factors include bone deficiencies, malalignment, ligamentous laxity, and muscle imbalance [2].

Rupture of MPFL typically occurs at the femoral origin, resulting in lateral dislocation with injury to the medial patella and lateral femoral condyle [3].

Modern MPFL reconstruction is a well-described operative intervention, which has been successful in a vast majority of young patients who have suffered from chronic patellar instability. Excellent functional results have been reported in skeletally immature patients at five-year follow-ups [3,4]. Despite a high rate of success, the combined complication and failure rate of MPFL reconstruction remains substantial in children and adolescents, reportedly at about 25% [4]. Recurrent instability, persistent knee pain, and loss of range of motion do occur in a subset of patients. Moreover, technical errors continue to be one of the major causes of complications and failures in MPFL reconstruction [5].

The purpose of conducting this systematic literature review was to analyze the data available concerning the complications and recurrence of instability following MPFL reconstruction for patellar instability in young and adolescent patients. Despite the good to excellent clinical outcomes reported in the literature about young and adolescent patients, there are no articles that have analyzed recurrence rate and complications after MPFL in young patients.

It was hypothesized that it is crucial for MPFL reconstruction surgery to restore the normal anatomy and isometry of the native ligament. Graft choice and methods of fixation play a secondary role in the achievement of successful outcomes.

## 2. Material and Methods

This systematic review was performed following the preferred reporting items for the systematic reviews (PRISMA) guidelines [6].

### 2.1. Eligibility Criteria

Studies from the existing literature in the English language were selected for this review based on the criteria outlined below.

### 2.2. Study Design

Studies that were conducted using randomized controlled trials (RCTs), controlled (non-randomized) clinical trials (CCTs), prospective and retrospective comparative cohort studies, case-control studies, and case series were included in this review. Case series involving less than 10 patients and/or those that did not report data on clinical and functional results were excluded.

### 2.3. Participants

Studies that involved children and adolescents (persons less than 20 years), living human subjects who underwent combined MPFL reconstruction, were considered eligible for the current study. Concomitant surgical procedures were not considered an exclusion criterion; however, MPFL repair was.

### 2.4. Interventions

Only those studies that reported on MPFL reconstruction as the primary intervention methodology were considered eligible to be included in this review. All of these studies that focused on clinical and functional outcomes were finally included.

### 2.5. Types of Outcome Measures

The outcome measures extracted from the reviewed studies were complications and recurrent patellar instability.

### 2.6. Information Sources and Search

A systematic literature search was carried out via the PubMed (MEDLINE), Scopus, EMBASE, and Cochrane Library databases. The date of publication was not considered an inclusion criterion, and the search was carried out in April 2021. Two independent reviewers (RD and PC) assisted in conducting and validating the search. The following terms were searched in the title, abstract, and keywords fields: “medial patellofemoral ligament” or “patellar instability/dislocation” and “reconstruction” and “children” or “adolescents” or “young patients.” Only those papers that were published in English were included. MPFL repair was considered as an exclusion criterion, and patients younger than 20 years at the time of their surgery were considered adolescents.

### 2.7. Data Collection and Analysis

#### Study Selection

The retrieved articles were first screened by title and then, if found to be relevant, screened further by abstract. After the exclusion of studies that did not meet the eligibility criteria, the entire contents of the remaining articles were evaluated for eligibility. To minimize the risk of bias, the authors reviewed and discussed all the selected articles and references as well as the articles excluded from the study. In the event of any disagreement between the reviewers, one of the senior investigators (NU) made the final decision. At the end of the process, a manual search was conducted for studies that might have been missed. This was done by going through the reference lists of the included studies and relevant systematic reviews.

### 2.8. Data Collection Process

Data were extracted from the included papers by the first two authors using a computerized tool created in Microsoft Access (Version 2010, Microsoft Corp, Redmont, WA, USA). Each article was validated once again by the first author before taking it up for analysis. For each of the studies, the data regarding the participants (age, gender, surgery and follow-up evaluation), the rates of complications, and recurrent patellar instability were extracted.

### 2.9. Levels of Evidence

The Oxford Levels of Evidence, as set by the Oxford Center for Evidence-Based Medicine, was used to categorize methodological quality. This tool classifies systematic RCTs and inception cohort studies as Level II evidence, cohort studies, or control arm of randomized trials as Level III evidence, and case series, case-control studies, or poor-quality prognostic cohort studies as Level IV evidence [7].

### 2.10. Evaluation of Study Quality

The quality of each selected study was evaluated by using the methodological index for the non-randomized studies (MINORS) score for which the following criteria were assessed: a clearly stated aim, the inclusion of consecutive patients, prospective data collection, endpoints appropriate to the aim of the study, unbiased assessment of the study endpoints, follow-up of less than 5%, prospective calculation of the study size, adequate control group, contemporary group, baseline group equivalence, and adequate statistical analysis [8]. The checklist included 12 items of which the last four were specific to the comparative studies. Each item was given a score of 0–2 points; the maximum possible score was set at 16 points for non-comparative studies and 24 points for comparative studies.

### 2.11. Statistical Analysis

Continuous variables were described using means and standard deviations or medians and ranges. Categorical variables were tabulated with absolute and relative frequencies.

## 3. Results

### 3.1. Search Results

**Figure 1:**
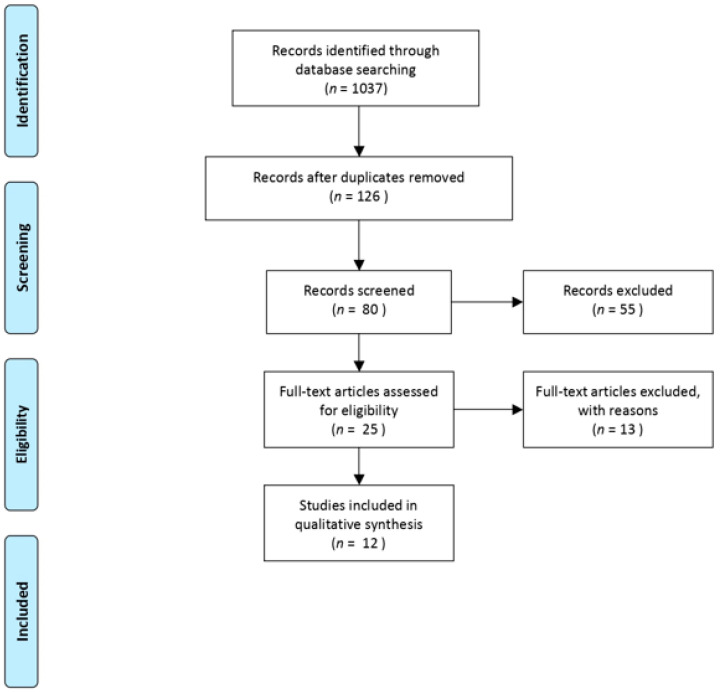
PRISMA flowchart showing the selection process of the papers.

The electronic search yielded 1037 studies. After 910 duplicates were removed, 126 studies remained, of which 80 were excluded after reviewing the abstracts, bringing down the number to 46. Of these, 13 articles were excluded after applying additional inclusion and exclusion criteria. No further studies were found from manually checking the reference lists of the selected articles. In the end, 12 articles that met all the inclusion criteria were finalized for our study [9,10,11,12,13,14,15,16,17,18,19,20]. The flowchart in Figure 1 depicts the selection process of the studies.

### 3.2. Patient Demographic

A total of 12 articles met the inclusion criteria. These were published from 2013 to 2019. Specific data in this regard are presented in Table 1. Of these 12 articles, seven were Level IV of evidence, three were Level III, and one each of Level II and Level I. In all, 332 patients were included in the review, of which 195 were female (63.5%) and 112 were male (36.5%), and the treated knees totaled 352. The mean age at the time of the surgery was 14.28 years, and the mean follow-up duration was 30.17 months. An autologous gracilis tendon was used in 185 (52.6%) cases; an allograft gracilis was used in 25 (7.1%) cases; the hamstring was not better specified in 44 (12.5%) cases; an adductor magnus was used in 32 (9.1%); an allograft fascia lata was used in 22 (6.3%); a pedicle quadriceps tendon was used in 25 (7.1%); a semitendinosus was used in 8 (2.3%); and the used graft was unspecified in 11 (3.1%) cases.

### 3.3. Outcomes of Interest

A total of 16 (4.5%) complications were reported: one (0.3%) patella fracture, one (0.3%) screw removal due to intolerance, one (0.3%) infection, five (1.4%) wound complications, six (1.7%) subluxations, and two (0.6%) instances of post-operative stiffness. A total of 18 (5.1%) recurrences of patellar instability were recorded.

### 3.4. Methodological Quality Assessment

The studies that were analyzed had a mean MINORS score of 10.75 (range 7–17), which confirmed the methodological quality of the available literature (Table 2).

## 4. Discussion

The most important finding of this systematic review was that the treatment of patellar instability with MPFL reconstruction in young patients is a safe and reliable procedure with a low risk of patellar dislocation recurrence (5.1%). The complications were also found to be minimal after MPFL reconstruction and can mostly be considered as minor complications as they do not influence the outcome of the patellar instability treatment.

A similar review was performed by Migliorini et al. to evaluate the treatments and outcomes of surgical management for recurrent patellar dislocations in skeletally immature patients.

The authors found that surgical procedures for skeletally immature patients affected by recurrent patellar dislocations are feasible and effective and post-operative complications and recurrence instability are infrequently. It is crucial to treat these patients as soon as possible to reduce the risk of new dislocation, to improve the level of sports activities, and especially to increase the quality of life (QOL) [21].

Patients with patellar dislocation or subluxation experience disabling symptoms with any activity, which consequently limits their recreational activity levels and adversely affects their QOL. In addition to the symptoms of instability, anterior knee pain is commonly overlaid in this population [22]. In particular, Hiemstra et al. found that a statistically significant correlation existed between measures of trochlear dysplasia and QOL physical symptom scores after an average of two years following patellofemoral stabilization surgery [23]. This statistically significant finding aligned with previous research that had demonstrated poorer clinical outcomes for MPFL imbrication and MPFL reconstruction in patients with high-grade trochlear dysplasia [23]. A similar finding in patients with patella alta was subsequently noted by the same author upon analyzing the QOL scores of patients with patella alta after they underwent MPFL reconstruction [24].

A high percentage of female patients (>60%) was found in the sample of patients analyzed; it has been well-recorded that females exhibit an increased incidence of generalized ligamentous laxity and anterior knee laxity compared to males [25]. Pfeiffer et al. analyzed which factors including sex were associated with increased rotatory knee laxity in collegiate athletes with no history of knee injuries; they confirmed that females were associated with increased rotatory knee laxity, measured during the pivot shift test and anterior translation as measured during the Lachman test. These findings confirmed that females are at a well-recognized risk for patella instability, especially in young patients [26].

The analysis of the reviewed data indicated that several graft types were used to perform MPFL reconstruction. Currently, in the literature, there is no agreement on the ideal graft for this surgery. Recently, Flanigan et al. determined the rate of recurrent dislocation and patellar instability following MPFL reconstruction with allograft or autograft tissue and compared the patient-reported outcomes for the patients who underwent allograft and autograft MPFL reconstruction [27]. A total of 87 patients with complete baseline data and a minimum one-year follow-up were analyzed, with a mean follow-up of 4.1 years after isolated MPFL reconstruction; Of these 57 and 30 patients had, respectively, undergone allograft and autograft reconstructions. In this regard, no significant differences were noted between the groups in terms of the sex and age of patients during reconstruction, their body mass index (BMI), or the time between follow-ups. Recurrent dislocation had occurred in two patients in the allograft group (3.5%) and in one patient in the autograft group (3.3%). Recurrent subjective instability had occurred in 17 patients in the allograft group (28.9%) and in 11 patients in the autograft group (36.7%) (n.s.). No significant differences were noted in the two groups as far as patient-reported outcomes were concerned [26].

Migliorini et al. performed a systematic review to identify and clarify the role of the gracilis and semitendinosus tendons as grafts for isolated MPFL reconstruction. Data from 1491 procedures were collected with a mean follow-up of 36.12 months. All the scores of interest (Kujala, Tegner, Lysholm) and range of motion were better in the semitendinosus group. Moreover, in favor of the semitendinosus group, a statistically significant reduction in revision surgeries and re-dislocations was evidenced. Additionally, the apprehension test and persistent instability sensation found statistical correlations [21].

McNeilan et al. conducted a systematic review and meta-analysis to determine whether graft selection affects patient outcomes after isolated MPFL reconstruction. The authors concluded that autograft was not superior to allograft or synthetic grafts for isolated reconstruction of the MPFL, and the rates of recurrent instability are generally low. Isolated MPFL reconstruction can provide significant symptom relief regardless of graft selection, though there is a bias toward reporting better-than-expected results among smaller studies. Pediatric patients and patients treated with adductor tendon autograft have higher recurrent instability rates [28].

Multiple studies have described MPFL reconstruction using anatomic and radiographic methods to obtain accurate tunnel placement [29,30,31]. Anatomic femoral and patellar tunnel placements are important in re-creating MPFL anatomy. Perhaps the most critical technical aspect of MPFL reconstruction is the anatomic femoral graft placement. Restoration of the true femoral insertion maintains isometry of the graft, which is essential for achieving favorable patella tracking throughout the knee’s range of motion [29,30,31]. Non-anatomic femoral insertion and the resulting graft anisometry have been associated with increased patellofemoral contact pressures in cadaveric studies as well as graft failures in reconstructions. The patellar insertion of the MPFL is a broad attachment along the proximal half of the medial patella and the distal-most vastus medialis obliquus (VMO). Graft placement onto this location has been associated with reduced patellar instability and low complication rates. Finally, a key role is played by the knee flexion angle in fixation, especially if there is a lack of consensus regarding the ideal knee flexion angle during MPFL fixation. The recommendations in the existing literature provide a wide range of descriptions, which present a challenge for surgeons seeking evidence-based guidance [32,33].

In skeletally immature patients, it is preferable not to create bone tunnels or dissect the periosteum in the region of the distal femoral physeal plate, which may require a modification of the femoral fixation methods being used [34]. Some of the techniques described are suitable for patients with open growth plates. These include the adductor magnus tendon transfer, adductor sling technique, and medial collateral ligament sling technique. It is important to remember that the medial femoral anatomic origin of the MPFL is distal to the physis. Due to the curved shape of the distal femoral physis, this can be confusing in the case of a lateral fluoroscopic view and appear proximal. However, the true location distal to the physis can be confirmed when it is viewed from the antero-posterior direction after selecting the Schottle’s point on the lateral [34,35,36]. The results of this systematic review confirmed that young patients, and those skeletally immature, can benefit from surgical MPFL reconstruction after patellar instability with a low rate of complications and new episodes of dislocations, regardless of the graft or the fixation used. This systematic review presents several limitations. First, the associated procedures were not considered for recurrence of instability; second, bone deformities were not taken into account; and third, the surgeons had used various types of grafts and fixation methods for different techniques based on their preference and experience. Additionally, the methodological quality of the selected studies must be noted. Almost all the studies were a retrospective case series of the mixed cohort and without a control group, with a low level of evidence and lack of randomization and blinding methods. Finally, the heterogeneity of the surgeries performed also represented a limitation of the study. Further studies should aim to clarify the role of each of the techniques and evaluate the potential superiority of a technique over the others. All these important methodological limitations highlight the need for additional well-designed prospective studies and further investigation of the issue.

## 5. Conclusions

MPFL reconstruction in young patients can be considered as an effective and safe treatment leading to clinical improvement. No major complications related to the technique were reported, but a high level of research evidence is required to better evaluate the clinical results in a long-term follow-up.

## Figures and Tables

**Table 1:** Demographic characteristics of the selected studies.

Author	Journal	Level of Evidence	Number of Patients	Mean Age (Years)	Graft Used for MPFL Reconstruction	Mean Follow-Up (Months)	Complications	Recurrence Instability
Uppstrom et al. (2019) [9]	KSSTA	IV—Case Series	49 patients—54 knees (30 F–19 M)	13.3 ± 1.6	54 Gracilis	28.8 (6–96)	None	5
Saper et al. (2019) [10]	Orthop J Sport Med	IV—Case Series	28 patients (20 F–8 M)	14.9 (12–16)	17 Hamstring11 Not specified	7.4	None	None
Fabricant et al. (2014) [11]	The Knee	IV—Case Series	27 patients (22 F–5 M)	14.9	27 Hamstring	3	None	None
Hohn et al. (2016) [12]	CORR	IV—Case Series	22 patients—25 knees (18 F–7 M)	16 ± 2.0	25 Gracilis	24 (12–44)	1 patella fracture1 1 screw removal due to inflammation	2
Lind et al. (2014) [13]	KSSTA	III—Case Control	20 patients—24 knees (11 F–9 M)	12.5	24 Gracilis	39 (17–72)	5 subluxation	4
Malecki et al. (2016) [14]	Int. Orthop.	III—Case Control	28 patients (32 knees)	14 (6–18)	32 Adductor Magnus	67.2 (36–180)	none	3
Matuszewski et al. (2018) [15]	Medicine	I—Randomized clinical trial	44 patients (27 F–17 M)	15 (13–17)	22 Fascia Lata Allograft 22 Gracilis	24	1 infection	1
Nelitz et al. (2013) [16]	AJSM	IV—Case Series	21 patients (6 F–15 M)	15 (14.4–16.4)	21 Gracilis	33.6 (24–43.2)	1 post-operative stiffness	None
Nelitz et al. (2017) [17]	KSSTA	III—Prospective Study	25 patients (16 F–9 M)	12.8 (9.5–14.7)	25 Pedicle Quadriceps Tendon	31.2 (24–40.8)	1 post-operative stiffness	None
Pesenti et al. (2018) [18]	Int. Orthop.	IV—Case Series	25 patients—27 knees (19–6 M)	13.8 ± 2.5	19 Gracilis 8 Semitendinosus	41.1 ± 13.5	5 wound complications	1
Roger et al. (2018) [19]	OTSR	II—Non-randomized prospective observational study	18 patients—20 knees (11 F–7 M)	14.6 (8–17)	20 Gracilis	38.7 (24–63)	None	None
Spang et al. (2019) [20]	J Ped Orthop	IV—Case Series	25 patients (15 F–10 M)	15.0 ± 2.2	25 Gracilis allograft	24 ± 6	1 subluxation	2

F = female; M = male; KSSTA = Knee Surgery Sports Traumatology Arthroscopy; CORR = Clinical Orthopedics and Related Research; AJSM = American Journal of Sports Medicine; OTSR = Orthopedics & Traumatol, Surgery & Research.

**Table 2:** MINORS score for the studies included in the review.

Author	A Clearly Stated aim	Inclusion of Consecutive Patients	Prospective Collection of Data	Endpoints Appropriate to the Aim of the Study	Unbiased Assessment of the Study Endpoint	Follow-up Period Appropriate to the Aim of the Study	Loss to Follow up Less Than 5%	Prospective Calculation of the Study Size	An Adequate Control Group	Contemporary Groups	Baseline Equivalence of Groups	Adequate Statistical Analyses	Total
									Additional criteria in the case of comparative study				
Uppstrom et al. (2019) [9]	**	*	*	**		**	**		n.a.	n.a.	n.a.	n.a.	10
Saper et al. (2019) [10]	**	*	*	**		*	**		n.a.	n.a.	n.a.	n.a.	9
Fabricant et al. (2014) [11]	**	*	*	*			**		n.a.	n.a.	n.a.	n.a.	7
Hohn et al. (2016) [12]	*	*	*	*		**	**		n.a.	n.a.	n.a.	n.a.	8
Lind et al. (2014) [13]	*	*	*	*		*	**		n.a.	n.a.	n.a.	n.a.	7
Malecki et al. (2016) [14]	**	*	**	**		*	**		**	**	**	*	17
Matuszewski et al. (2018) [15]	**	*	**	**		**	**		**	**	*	*	16
Nelitz et al. (2013) [16]	**	**	**	**		**	**		n.a.	n.a.	n.a.	n.a.	12
Nelitz et al. (2017) [17]	**	**	**	**		**	**		n.a.	n.a.	n.a.	n.a.	12
Pesenti et al. (2018) [18]	**	*	*	**		*	**		n.a.	n.a.	n.a.	n.a.	9
Roger et al. (2018) [19]	**	**	**	**		*	**		n.a.	n.a.	n.a.	n.a.	11
Spang et al. (2019) [20]	**	*	*	**		**	**		n.a.	n.a.	n.a.	n.a.	11

The items were scored 0 (not reported), * (reported but inadequate), or ** (reported and adequate). The global ideal score was 16 for non-comparative studies and 24 for comparative studies. n.a. = not available.
